# An adaptive categorical effect size method based on intuitionistic meta fuzzy functions

**DOI:** 10.1038/s41598-023-44691-6

**Published:** 2023-10-13

**Authors:** Ayşegül Yabacı Tak

**Affiliations:** Department of Biostatistics and Medical Informatics, Faculty of Medicine, Bezmialem Vakıf University, Istanbul, Türkiye

**Keywords:** Scientific data, Software, Statistics, Outcomes research, Preclinical research

## Abstract

There are several categorical effect size methods in the literature. It is not clear which method performs better for a given dataset and it is a challenging task to select the correct method for a given dataset. In this sense, to overcome the questions like “Which method should we choose?” and “Which categorical effect size method is more reliable for a given dataset?”, an adaptive categorical effect size method based on intuitionistic meta fuzzy functions is introduced in the paper. Thus, the main motivation of the proposed method is to obtain more accurate outcomes by combining the results of better performing methods instead of relying on only one method. In the study, the intuitionistic fuzzy c-means clustering algorithm is adapted to meta fuzzy functions by incorporating not only membership degrees but also non-membership degrees to improve the clustering accuracy of meta fuzzy functions. Meta fuzzy functions are the linear combination of seven categorical effect size methods and the weights, which are calculated from membership grades from intuitionistic fuzzy c-means algorithm. Among the functions, the one with the lowest mean absolute percentage error is selected as the best. To evaluate the performance of the proposed method, *2 × 3, 2 × 4*, and *3 × 4* contingency tables were simulated. Additionally, the performance of the proposed method is also assessed by applying it to a real-time dataset. Experimental results show that the proposed method outperforms compared to the evaluated seven categorical effect size methods in terms of mean absolute percentage error. Also, the calculated effect sizes are within the range of ±10% in terms of bias. Thus, the results verified that proposed method achieves greater reliability.

## Introduction

Statistical significance (p-value) is the probability that the observed difference between two groups is due to chance. If the p-value is greater than the chosen alpha level, it is assumed that any observed difference can be explained by the variability of the sample size. When conducting statistical comparisons with exceptionally large sample sizes, it is highly likely that the p-value will consistently indicate a significant difference. However, statistically significant differences that arise due to the large number of data points do not always represent meaningful differences in reality^1^. A statistically significant result may sometimes arise simply from using a large sample. Statistical significance depends on both the sample size and effect size (ES) but the effect size is generally independent of the sample size^2^. Therefore, reporting only the p-value, especially in large samples, is not sufficient for readers to fully understand the implications^3,4^. Effect size (ES) is substantial of quantitative research, and it indicates the real magnitude of the effect. In addition to statistical significance, it enables researchers to understand the practical significance of the findings. Statistical hypothesis tests can be misleading due to type 1 and type 2 errors made depending on the sample size. For this reason, it is necessary to report the effect size as well as the p-value in many disciplines. The seven categorical effect size methods, which is used for $r\times c$ *c*ontingency tables in statistics in the study, are explained in section “Categorical effect size methods”. The $Cramer{^\prime}s V$ effect size measure has some disadvantages. First, $Cramer{^\prime}s V$ is a symmetric measure of association^5–7^. Second, it is zero under the assumption of independence. Third, interpretation of $Crame{r}{\prime}s V$ effect size measures is difficult^8^. $Tschuprow{^\prime}s T$ measure is closely related to $Cramer{^\prime}s V$ measure but less well-known^9^. Since it is a simple function of the Pearson chi-square statistic, it is among the commonly used effect sizes. Barely, the bias of the measure is large in data with small samples and it is difficult to interpret^8^. $Cohen{^\prime}s w$ is more appropriate for larger contingency tables^10^. $Uncertainty coefficient (U)$ is also commonly used effect size to measure the validity of a statistical classification algorithm^11^.

Considering the disadvantages of the ES methods, it is important to select the correct ES method for a given dataset. To overcome the aforementioned disadvantages, selected 7 ES methods are aggregated in functions based on their performances for a given dataset. In this sense, the motivation of this paper is to combine different categorical effect sizes methods in functions with Meta Fuzzy Functions $(MFF)$ based on Intuitionistic Fuzzy C-Means Clustering $\left(IFCM\right)$ algorithm. Fuzzy c-means (*FCM*) clustering algorithm is used in *MFF*. *FCM*, proposed by Bezdek et al.^12^, stands out as one of the frequently employed methods because of its simplicity and the benefits it offers compared to the k-means clustering algorithm. Nevertheless, it has certain drawbacks, including its susceptibility to initial settings and sensitivity to noise. In this sense, *IFCM* that accounts for hesitancy of an object belonging to a cluster is employed in *MFF*. Intuitionistic Fuzzy Sets $(IFSs)$ are introduced as a modification of Zadeh’s fuzzy set theory by Atanassov^13,14^. The main difference between fuzzy sets and $IFSs$ is that fuzzy sets only consider membership degree while $IFSs$ consider both membership and non-membership degrees. That is, *IFSs* account also for the hesitancy of membership grades in clusters. Thus, the centers of the clusters are obtained more accurately. It has been determined by the studies that IFSs are more effective than traditional fuzzy set theory by overcoming uncertainty^15^. $IFSs$ have been commonly used for forecasting and engineering problems. In addition to time series and forecasting methods, $IFSs$ are widely used in the field of medicine for clustering images and diagnostics^16–18^. Numerous studies employing *IFSs* have been proposed by Fan et al.^19^, Kumar and Gangwar^20^, Lei, et al.^21^, Tak^22^, Gwak et al.^23^.

Because aforementioned advantages of *IFCM* in the literature, it is employed in *MFF*. The $MFF$ was proposed by Tak^24^. The purpose of the *MFF* is to combine methods or definitions used for the same purpose. Its logic is simply based on meta-analysis. Meta-analysis is a method that combines the outcomes of multiple studies to yield stronger results for a specific purpose. For example, Tak and Gök^25^ and Gök and Tak^26^ utilized the *MFF* to merge different definitions of currency crisis. By employing this approach, they aimed to enhance the accuracy and reliability of their analysis. Similarly, Tak et al.^27^ employed the *MFF* to combine various time series methods. Their objective was to improve the forecasting performance by integrating multiple forecasting techniques within the framework. Cevik et al.^28^ used the *MFF* approach to forecast the number of immigrants within the maritime line. Tak^29^ used the *MFF* approach to forecast combination. These studies have shown that combining different methods with the $MFF$ has better estimation accuracy.

Yabacı Tak and Ercan^30^ ensembled some ES definitions for two independent groups with *MFF* to obtain a more accurate effect size value. Yabacı Tak and Ercan^30^ combined six effect size methods for numerical variables with the *MFF* approach by using classical fuzzy c-means algorithm $(FCM),$ which can be used with or without the assumption of normal distribution. The combined methods in the previous study were not used for categorical variables. Thus, numerous categorical ES methods are combined in this study. Besides, the $FCM$ clustering method only uses membership degrees while calculating the cluster centers. Thus, the $MFF$ approach with the $IFCM$, which provides a more accurate estimation of the cluster centers, has been developed in the study.

In the light of this information, we will introduce intuitionistic meta fuzzy categorical effect size functions $\left( {I - MFCESF} \right)$ approach. The aim of the study is to obtain better outcomes by combining seven categorical effect size measures in functions. The purpose of combining the ES is the assumption that each measure might have much or partial information for a given dataset. Therefore, while the methods that perform better will be gathered into one function, the methods that perform worse will be gathered into another. In the remainder of the paper, we will describe the *IFCM* and the meta fuzzy functions briefly in the section “Preliminaries”. The proposed method $\left(I-MFCESF\right)$ is discussed in section “Intuitionistic meta fuzzy categorical effect size functions (1-MFCESF)”. The performance of the proposed method is evaluated with some applications for simulated and real datasets in section “Evaluation”. Finally, the results of the proposed method are discussed in section “Conclusion”.

## Preliminaries

The methods (effect sizes, intuitionistic fuzzy c-means and meta fuzzy function) that are used in the paper are detailed in this section.

### Categorical effect size methods

Short descriptions of seven types of ES measures are provided for $r\times c$ contingency tables. $Cramer{\prime}s V$ is proposed in 1946 and it is an effect size measure that is generally used with nominal variables in $r\times c$ contingency tables^7,31–34^. It is calculated in Eq. (1) based on Pearson’s chi-square statistic. It takes values between 0 and + 1.

1 $$V = \sqrt {\frac{{\varphi^{2} }}{{\min \left( {c - 1,r - 1} \right)}}} = \sqrt {\frac{{{\raise0.7ex\hbox{${\chi^{2} }$} \!\mathord{\left/ {\vphantom {{\chi^{2} } n}}\right.\kern-0pt} \!\lower0.7ex\hbox{$n$}}}}{{\min \left( {c - 1,r - 1} \right)}} }$$

where, ${\chi }^{2}$ is the Pearson’s chi-squared statistics, $n$ is the total observations number, $c$ is the number of coloumns and $r$ is the number of rows. In the Eq. (1), numerator of formula is based on the observed frequencies, denominator of formula is based on an unobserved frequencies. Therefore, when $Crame{r}{\prime}s V=1$, the marginal frequencies are not zero and *r* or *c* has not zero cell frequencies.

$Tschuprow{^\prime}s T$ is a ES which measures the association between two nominal variables in $rxc$ contingency tables^35^. It takes values between 0 and +1, and calculated in Eq. (2).

2 $$T = \sqrt {\frac{{\varphi^{2} }}{{\left( {c - 1} \right) \times \left( {r - 1} \right)}}}$$

where, ${\chi }^{2}$ is the Pearson’s chi-squared statistics, $c$ is the number of coloumns and $r$ is the number of rows.

Another measure of categorical effect size is the $Pearson{^\prime}s contingency coefficient$ ($Pearson{^\prime} s c$). It takes values between 0 and + 1. $Pearson{^\prime} s c$ can be calculated in Eq. (3)^36^.

3 $$Pearson{^\prime}s c = \sqrt {\frac{{\chi^{2} }}{{\chi^{2} + n}}}$$

where, $\chi^{2}$ is the Pearson’s chi-squared statistics, and* n* is the total number of observations.

$Cohen{^\prime} s w$ effect size is proposed by Cohen^37^.$Cohen{^\prime} s w$ should be used for larger contingency tables. Cohen’s w effect size measure is obtained in Eq. (4).

4 $$w = \sqrt {\mathop \sum \limits_{i = 1}^{m} \frac{{\left( {p_{1i} - p_{0i} } \right)^{2} }}{{p_{0i} }}}$$

where,* m* is the number of cells, $p_{0i}$ is the value of the ith cell under the null hypothesis, $p_{1i}$ is the value of the ith cell under the alternative hypothesis.

$Goodman - Kruskal Tau \left( {G - K Tau} \right)$ is another ES measure of nominal variables. It measures the predictability of the column or row variable given the value of other variables, in percentage. The measure varies between 0 and 1^38,39^. $G - K Tau$ is calculated in Eq.(5)^40^.

5 $$GK - Tau = \frac{{n\mathop \sum \nolimits_{ij} \left( {\frac{{a_{ij}^{2} }}{{a_{.j} }}} \right) - \mathop \sum \nolimits_{i} a_{i.}^{2} }}{{n^{2} - \mathop \sum \nolimits_{i} a_{i.}^{2} }} , \;\; i = 1, \ldots r,\;\; j = 1, \ldots c$$

where, $n$ is the total number of observation, $a_{ij}$ is the value of number of observation in ith row and jth column, $a_{.j}$ is the total number of observation in jth column and $a_{i.}$ is the total number of observation in ith row.

$Uncertainty coefficient \left( U \right)$ is first introduced by Theil^41^. It is also called Proficiency, Entropy Coefficient or Theil’s U. It is often used as a measure of the ES of nominal variables in statistics and takes the value between 0 and + 1. This measure is defined in Eq. (6)

6 $$U\left( {XY} \right) = \frac{{H\left( X \right) - H\left( {XY} \right)}}{H\left( X \right)} = \frac{{I\left( {X;Y} \right)}}{H\left( X \right)}$$

7 $$H\left( X \right) = - \mathop \sum \limits_{x} P_{X} \left( x \right)logP_{X} \left( x \right) , H\left( {XY} \right) = - \mathop \sum \limits_{x,y} P_{X,Y} \left( {x,y} \right)logP_{{X \rm{\mid }Y}} \left( {x \rm{\mid }y} \right)$$

where, $H\left( X \right)$ is the entropy of a single distribution, $H\left( {XY} \right)$ is the conditional entropy and $U\left( {XY} \right)$ is the uncertainty coefficient. $P_{X,Y} \left( {x,y} \right)$ is the conditional distribution.

$Goodman - Kruskal Lambda \left( \lambda \right)$ statistic is an effect size proposed to measure the strength of the relationship between two nominal variables by evaluating the proportional reduction of error (PRE)^39^. Also, $\lambda$ is the asymmetrical measure. The $\lambda$ statistic takes value between 0 and 1. How to calculate the $\lambda$ statistic is given in Eq. (8).

8 $$\lambda = \frac{{E_{1} - E_{2 } }}{{E_{1} }}$$

where, $E_{1}$ is the number of prediction errors made when the independent variable is ignored, $E_{2}$ equal to the number of prediction errors made when the prediction is based on the independent variable.

### IFCM

Over the past decades, the fuzzy set theory proposed by Zadeh^14^ has been expanded with different approaches. Among these, intuitionistic fuzzy set theory, which has been commonly used in the literature and has many applications in different fields, was developed by Atanassov^13^. While only the membership degree is taken into account in the FCM, non-membership degree is also taken into account in IFCM. So that, the centers of the clusters are calculated more accurately. Algorithm are given below^22^:

Step-1.: Determine the number of clusters $\left( c \right)$, the fuzziness index (*f*), and initialize the cluster centers $\left( {v_{i} } \right)$ randomly.
Step-2.: Calculate the degrees of membership ($\mu$) and non-membership ($u$). Formulas are given in Eqs. (9–11):
  9 $$\mu_{ik} = \left[ {\mathop \sum \limits_{j = 1}^{c} \left( {\frac{{d\left( {x_{k} ,v_{i} } \right)}}{{d\left( {x_{k} ,v_{j} } \right)}}} \right)^{{\frac{2}{f - 1}}} } \right]^{ - 1} , \;\;i = 1,2, \ldots ,c ;\;\;k = 1,2, \ldots ,n$$
  where $d\left( . \right)$ is the Euclidean distance between *k*th data in the *i*th cluster center:
  10 $$u_{ik} = \left( {1 - \mu_{ik}^{\alpha } } \right)^{{{\raise0.7ex\hbox{$1$} \!\mathord{\left/ {\vphantom {1 \alpha }}\right.\kern-0pt} \!\lower0.7ex\hbox{$\alpha $}}}} , \alpha > 0$$
  11 $$\mu_{ik}^{*} = 1 - u_{ik}$$
Step-3.: Update the cluster centers by using Eq. (12):
  12 $$v_{i} = \frac{{\mathop \sum \nolimits_{k = 1}^{n} \left( {\mu_{ik}^{*} } \right)^{f} x_{k} }}{{\mathop \sum \nolimits_{k = 1}^{n} \left( {\mu_{ik}^{*} } \right)^{f} }} , i = 1,2, \ldots ,c$$
Step-4.: Algorithm is ended if the difference between two iterations are dropped under some given threshold ε; otherwise, repeated Step-2 and Step-3.

### Meta fuzzy functions

Tak^24^ proposes *MFF* to combine different methods or definitions, such as prediction and forecasting. The *MFF* consists of three components: functions, weights, and the best meta fuzzy function. Functions; the linear combination of weights and the findings of the selected methods. Weights: the membership grades that are obtained from *FCM* clustering algorithm are used to compute weights. The best meta fuzzy function: the function that has the best evaluation criteria. Meta fuzzy functions begin with obtaining the outcomes of the methods chosen for a purpose as the input matrix. After that, the input matrix is clustered using fuzzy c-means clustering algorithm to separate the categorical ES methods based on how well they predict outcomes. As a result, each method will be assigned to a cluster with a membership grade. Then, using membership grades for each cluster, the weights of the methods are calculated. In this case, there will be an equal number of functions as the cluster number. Finally, the best meta fuzzy function is selected based on its evaluation criteria.

## Intuitionistic meta fuzzy categorical effect size functions $\left( {{\varvec{I}} - {\varvec{MFCESF}}} \right)$

$Cramer{^\prime} s v, \;\;Tschuprow{^\prime} s T, \;\;Pearson{^\prime} s c, \;\;Cohen{^\prime} s w,\;\; G - K Tau,\;\; U$ and $\lambda$ methods can be used to calculate effect size measures for a dataset. However, there is no definite information in the literature about which method is better or in which situations it should be used. Therefore, the performance of the methods may change according to the type of datasets. Because the performance of the ES measures in the proposed method is uncertain, we are looking for the optimum weights of the ES measures in the combination function. For this purpose, $I - MFCESF$ method is proposed in this paper. The ES measures are clustered based on their performances by using the *IFCM*. There will be as many functions as the number of clusters. Functions are obtained by multiplying each method by its weight in the clusters. The ES measures that perform better for the dataset will be in a function with a higher membership degree, while the ES measures that perform worse will be in another function with a higher degree of membership. Finally, the function with the minimum model evaluation criterion is selected as $I - MFCESF _{best}$ and new effect size value will be calculated for the dataset. So, $I - MFCESF$ method is an adaptive combination of categorical effect size measures. Step-by-step algorithm, pseudocode and flowchart are given below for $I - MFCESF$ approach.

**Algorithm 1**

Step 1.: Determine $m$ categorical ES measures and simulated data randomly for *t* iterations. Obtain input matrix *(Z)* by applying $m$ measures to the simulated dataset for *t* repeats.
  13 $$Z = [Z_{ij} ] , i = 1,2, \ldots ,t ;\;\; j = 1,2, \ldots ,m$$
  where, $Z_{ij}$ is the ES value of *i*th repeat for *j*th measure.
  $$Z = \left[ {\begin{array}{*{20}c} {Z_{1,1} } & {Z_{1,2} } & \ldots & {Z_{1,t} } \\ {Z_{2,1} } & {Z_{2,2} } & \ldots & {Z_{2,t} } \\ \vdots & \vdots & \ldots & \vdots \\ {Z_{m,1} } & {Z_{m,2} } & \ldots & {Z_{m,t} } \\ \end{array} } \right]$$
Step 2.: The input matrix is clustered by using intuitionistic fuzzy c-means.
  Step 2.1.: The number of fuzzy clusters $\left( c \right)$ is determined and fuzzy index value $\left( f \right)$ and center of clusters $\left( v \right)$ are initialized.
  Step 2.2.: The degrees of membership ($\mu$) and non-membership value are calculated in each cluster with Eqs. (9–11).
Step 2.3.: The new clusters center is calculated by using Eq. (12).
Step 2.4.: If the difference between two iterations drops under some threshold, stop the algorithm; otherwise, repeat Step 1 and Step 2.

Step 3.: Intuitionistic meta categorical effect size functions are obtained. $I - MFCESF$ is given in Eq. (14).
  14 $$I - MFCESF_{i} \left( z \right) = \mathop \sum \limits_{j = 1}^{m} w_{ij} z_{j} , \;\; i = 1,2, \ldots ,c$$
  15 $$w_{ij} = \frac{{\mu_{ij}^{*} }}{{\mathop \sum \nolimits_{j = 1}^{m} \mu_{ij}^{*} }} ,\; i = 1,2, \ldots , c$$
  where, c is the number of clusters, $\mu_{ij}^{*}$ is the membership grades of *j*th method in $i$th cluster,$I - MFCESF_{i}$ is the *i*th intuitionistic meta categorical effect size functions, and $w_{ij}$ is weight of *j*.th method in $i$th cluster.
Step 4.: Select the best intuitionistic meta categorical effect size functions that has the minimum Mean absolute percentage error (MAPE).

**Figure 1:**
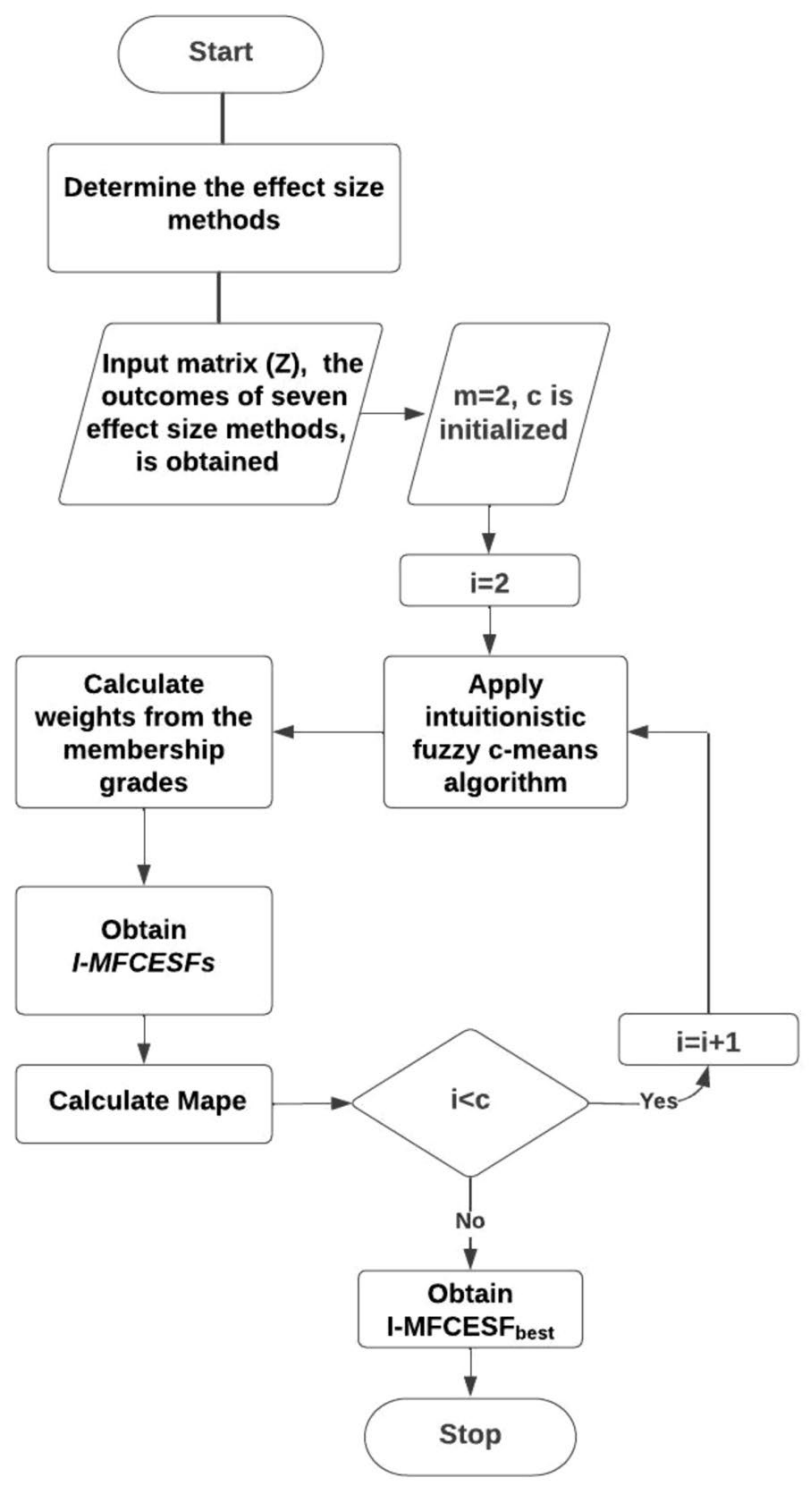
Flowchart of $$I-MFCESF$$


MAPE values are calculated for select $I - MFCESF_{best}$. Mape formula is given in Eq. (16).

16 $$MAPE = \frac{1}{n}\mathop \sum \limits_{i = 1}^{n} \left| {\frac{{y_{i} - \hat{y}_{i} }}{{y_{i} }}} \right|$$

where, $y_{i }$ is the mean of the ES value calculated from each method for the population and $\widehat{{y_{i} }}$ is the predicted ES value obtained from 1000 simulated samples. The pseudo code and the flow chart of $I - MFCESF$ based on *MFF* is given Algorithm 2 and Fig. 1, respectively.

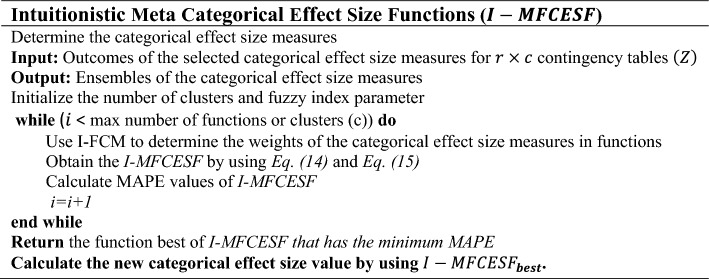


## Evaluation

The estimation performance of the proposed I-MFCESF method is evaluated through both simulation studies and the use of real-world datasets. In the simulation study, random generation of two categorical variables (x and y) is performed to create contingency tables of different sizes (2 × 3, 2 × 4, and 3 × 4). These tables are generated for a sample size of *N = 1000* and repeated for *t = 1000* iterations. Real-world datasets are obtained from the UCI Machine Learning Repository^42^, and 1000 different samples are taken with replacement from these datasets. By applying the selected categorical effect size methods to each dataset, an input matrix (Z) is obtained. The *I-MFCESF* method incorporates two crucial parameters: the number of clusters (c) and the fuzziness index parameter (m). To determine the optimal number of clusters (c), the minimum mean absolute percentage error (*MAPE*) for the *I-MFCESF* is calculated iteratively between 2 and 5. Due to the lack of consensus on the optimal value for the fuzziness index parameter of *IFCM* (intuitionistic fuzzy c-means algorithm), a value of 2 is selected for this study. The performance of the proposed method is evaluated using the *MAPE*, which measures the average percentage difference between the estimated values and the true values.

The simulation study and real-world dataset applications of the *I-MFCESF* method are conducted using R Studio. Various R package namely “ppclust,” “effectsize,” “DescTools,” “fclust,” “rcompanion, ” “remotes,” “githubinstall,” and “Metrics, ” are utilized^43–47^. As an application, seven different selected categorical ES measures are combined by using the *MFF* based on the intuitionistic fuzzy c-means to obtain more accurate results for all datasets.

### Simulated 2 $\times$ 3, 2 $\times 4$ and 3 $\times$ 4 contingency tables for the datasets of categorical variables

Two categorical variables *x* and y ($2 \times 3$, $2 \times 4$ and $3 \times 4$ contingency tables) are simulated randomly for *N *= 1000 sample size and *t *= 1000 iterations. Selected measures: $Cramer{^\prime} s v$ (Metasure 1), $Tschuprow{^\prime} s T$ (Measure 2), $Pearson{^\prime} s c$(Measure 3), $Cohen{^\prime} s w$ (Measure 4), $G - K Tau$(Measure 5), $U$ (Measure 6) and $\lambda$ (Measure 7) are applied to all datasets. The input matrix $\left( Z \right)$ consists of the outcomes of the ES measures for the simulated data set. The proposed method utilizes the *IFCM* clustering algorithm, where the fuzziness index parameter (m) is set to 2. After obtaining the input matrix, the *IFCM* algorithm is applied. In this method, the number of functions is equal to the optimal number of clusters. Functions are obtained by multiplying the weights of the methods with the actual value and sum them (Eq. 14) up. The weights of each method in each function are obtained as in Algorithm 1 (Step 3). Finally, the *MAPE* values are calculated for each from obtained $I - MFCESF$ functions. When calculating the *MAPE* values, the actual value is considered as the average of the values calculated from the dataset of the selected seven ES measures. The function with the lowest Mean Absolute Percentage Error is chosen as $I - MFCESF_{best }$ and the new ES value is computed based on this selection.

The first dataset is simulated for 2 $\times$ 3 contingency table and the input matrix ($Z$) is obtained by applying the selected categorical ES methods. The first five and last five prediction values of the input matrix are summarized in Table 1.

**Table 1:** Input Matrix for 2 $\times$ 3 contingency table

[Z]	t_1_	t_2_	t_3_	t_4_	t_5_		t_996_	t_997_	t_998_	t_999_	t_1000_
*Cramer’s v*	0.0382	0.0353	0.0507	0.0223	0.0112	…	0.0350	0.0215	0.0363	0.0202	0.0266
*Pearson’s c*	0.0587	0.0569	0.0674	0.0499	0.0460	…	0.0567	0.0496	0.0575	0.0490	0.0519
*Tschuprow’s T*	0.0495	0.0479	0.0568	0.0420	0.0387	…	0.0477	0.0417	0.0484	0.0412	0.0437
*Cohen’s w*	0.0588	0.0570	0.0676	0.0500	0.0461	…	0.0568	0.0496	0.0576	0.0491	0.0520
*G-K Tau*	0.0034	0.0032	0.0045	0.0025	0.0021	…	0.0032	0.0024	0.0033	0.0024	0.0027
*U*	0.0019	0.0018	0.0025	0.0013	0.0011	…	0.0018	0.0013	0.0018	0.0013	0.0015
$\lambda$	0.0175	0.0304	0.0152	0.0222	0.0217	…	0.0225	0.0176	0.0194	0.0018	0.0220

For the first simulated dataset, the optimal cluster number, which is set to 2, is determined by selecting the minimum *MAPE* value for $I-MFCESFs$. As a result, two functions are obtained by multiplying each method with their respective weights. The weights for the $I-MFCESF$ are computed using intuitionistic membership grades, as outlined in Table 2. The functions of the proposed method are obtained using the following equations (Eqs. 17, 18).

17 $$\begin{aligned} I - MFCESF_{1 } & = Cramer^{\prime}s v \times 0.2117 + Pearson^{\prime}s c \times 0.2352 + Tschuprows^{\prime}T \times 0.2388 \\ & \;\;\; + Cohen^{\prime}s w \times 0.2349 + G - K Tau \times 0.0082 + U \times 0.0133 \\ & \;\;\; + \lambda \times 0.0578 \\ \end{aligned}$$

18 $$\begin{aligned} I - MFCESF_{2 } & = Cramer^{\prime}s v \times 0.0866 + Pearson^{\prime}s c \times 0.0143 + Tschuprows^{\prime}T \times 0.0057 \\ & \;\;\; + Cohen^{\prime}s w \times 0.0150 + G - K Tau \times 0.3022 + U \times 0.2985 \\ & \;\;\; + \lambda \times 0.2777 \\ \end{aligned}$$

**Table 2:** Weights of the $\mathrm{I}-\mathrm{MFCESF}$ for 2 $\times$ 3 contingency table

	${I-MFCESF}_{1}$	${I-MFCESF}_{2}$
$Cramer{^\prime}s v$	0.2117	0.0866
$Pearson{^\prime}s c$	0.2352	0.0143
$Tschuprows{^\prime}T$	0.2388	0.0057
$Cohen{^\prime}s w$	0.2349	0.0150
$G-K Tau$	0.0082	0.3022
$U$	0.0133	0.2985
$\lambda$	0.0578	0.2777
MAPE	0.8126	**0.4168**

Significant values are in [bold].

Table 2 provides a clear depiction that $I - MFCESF_{2 }$ exhibits the lowest *MAPE.* Therefore, $I - MFCESF_{2 }$ is identified as the best *I-MFCESF*. The *MAPE* values are computed and presented in Table 3, to assess the performance of the proposed method.

**Table 3:** MAPE and BİAS values of the proposed and selected effect size methods for 2 $\times$ 3 contingency table

Categorical effect size methods	*MAPE*	*BİAS*
$Cramer{^\prime}s v$	0.9021	− 0.0559
$Pearson{^\prime}s c$	0.8455	− 0.083
$Tschuprows{^\prime}T$	0.8171	− 0.068
$Cohen{^\prime}s w$	0.8462	− 0.0835
$G-K Tau$	0.8896	0.0038
$U$	2.1825	0.0083
$\lambda$	0.7056	− 0.0238
*I-MFCESF*	**0.4168**	− **0.0106**

Significant values are in [bold].

Table 3 clear that the *I-MFCESF* outperforms the other categorical ES methods in terms of the *MAPE* values. According to the Li et al.^48^ a parameter prediction is considered acceptable when the bias is within ± 10%. The bias value of the proposed method was determined as − 1% in Table 3. Thus, the accuracy of the method is also sufficient in terms of bias.

A subsequent dataset is simulated for a 2 × 4 contingency table, and the input matrix (Z) is obtained by applying the chosen categorical ES methods. Table 4 provides a summary of the first five and last five prediction values found in the input matrix.

**Table 4:** Input matrix for 2 $\times$ 4 contingency table

[Z]	t_1_	t_2_	t_3_	t_4_	t_5_		t_996_	t_997_	t_998_	t_999_	t_1000_
*Cramer’s v*	0.1029	0.1116	0.0593	0.0247	0.1466	…	0.0635	0.0895	0.0762	0.0642	0.0497
*Pearson’s c*	0.1277	0.1346	0.0971	0.0811	0.1635	…	0.0997	0.1175	0.1080	0.1001	0.0917
*Tschuprow’s T*	0.0978	0.1032	0.0741	0.0618	0.1259	…	0.0761	0.0899	0.0826	0.0764	0.0699
*Cohen’s w*	0.1288	0.1358	0.0976	0.0813	0.1658	…	0.1002	0.1184	0.1087	0.1006	0.0920
*G-K Tau*	0.0165	0.0184	0.0095	0.0066	0.0274	…	0.0100	0.0140	0.0118	0.0101	0.0084
*U*	0.0081	0.0090	0.0046	0.0032	0.0134	…	0.0048	0.0067	0.0057	0.0049	0.0041
$\lambda$	0.0183	0.0369	0.0296	0.0087	0.0309	…	0.0354	0.0447	0.0202	0.0220	0.0302

The weights for the $I-MFCESF$ are calculated by using intuitionistic membership grades as in Table 5, and the functions of the proposed method are obtained as in Eqs. (19, 20).

19 $$\begin{aligned} I - MFCESF_{1 } & = Cramer^{\prime}s v \times 0.239 + Pearson^{\prime}s c \times 0.2386 + Tschuprows^{\prime}T \times 0.2335 \\ & \;\;\; + Cohen^{\prime}s w \times 0.2380 + G - K Tau \times 0.0039 + U \times 0.0116 \\ & \;\;\; + \lambda \times 0.0395 \\ \end{aligned}$$

20 $$\begin{aligned} I - MFCESF_{2 } & = I - MFCESF_{best } = Cramer^{\prime}s v \times 0.0386 + Pearson^{\prime}s c \times 0.0144 \\ & \;\;\; + Tschuprows^{\prime}T \times 0.0252 + Cohen^{\prime}s w \times 0.0156 + G \\ & \;\;\; - K Tau \times 0.3088 + U \times 0.3036 + \lambda \times 0.2938 \\ \end{aligned}$$

**Table 5:** Weights of the $\mathrm{I}-\mathrm{MFCESF}$ for 2 $\times$ 4 contingency table

Categorical effect size methods	2 $\times 4$	
	${I-MFCESF}_{1}$	${I-MFCESF}_{2}$
$Cramer{^\prime}s v$	0.2349	0.0386
$Pearson{^\prime}s c$	0.2386	0.0144
$Tschuprows{^\prime}T$	0.2335	0.0252
$Cohen{^\prime}s w$	0.2380	0.0156
$G-K Tau$	0.0039	0.3088
$U$	0.0116	0.3036
$\lambda$	0.0395	0.2938
*MAPE*	0.7012	**0.3581**

Significant values are in [bold].

Table 5 clearly shows ${I-MFCESF}_{2 }$ has the lowest *MAPE.* Thus, the best *I-MFCESF* is ${I-MFCESF}_{2}$. The *MAPE* values of the methods are computed, and the results are presented in Table 6 to assess the performance of the proposed method.

**Table 6:** MAPE and BİAS values of the proposed and selected effect size methods for $2\times 4$ contingency tables

Categorical effect size methods	2 $\times 4$	
	*MAPE*	*BİAS*
$Cramer{^\prime}s v$	0.6587	− 0.0750
$Pearson{^\prime}s c$	0.7491	− 0.1016
$Tschuprows{^\prime}T$	0.6725	− 0.0707
$Cohen{^\prime}s w$	0.7512	− 0.1030
$G-K Tau$	1.1339	0.0122
$U$	3.3170	0.0221
$\lambda$	0.5845	− 0.0157
*I-MFCESF*	**0.3581**	− **0.0019**

Significant values are in [bold].

Based on the information provided in Table 6, it is evident that the *I-MFCESF* method demonstrates superior performance compared to the individual categorical effect size methods in terms of *MAPE*. The bias of the proposed method is determined as respectively − 1.9%. Because bias is between ± 10%, the accuracy of the proposed method is also sufficient in terms of bias.

Lastly, a dataset is simulated for a 3 × 4 contingency table, and the input matrix (Z) is generated by applying the selected categorical ES methods. Table 7 provides a summary of the first five and last five prediction values found in the input matrix.

**Table 7:** Input Matrix for 3 $\times$ 4 contingency table

[Z]	t_1_	t_2_	t_3_	t_4_	t_5_		t_996_	t_997_	t_998_	t_999_	t_1000_
*Cramer’s v*	0.0431	0.0212	0.0793	0.0413	0.0344	…	0.0376	0.0449	0.0343	0.0301	0.0152
*Pearson’s c*	0.0981	0.0828	0.1349	0.0965	0.0911	…	0.0935	0.0997	0.0910	0.0881	0.0801
*Tschuprow’s T*	0.0630	0.0530	0.0870	0.0620	0.0584	…	0.0600	0.0640	0.0584	0.0565	0.0513
*Cohen’s w*	0.0986	0.0831	0.1362	0.0970	0.0915	…	0.0940	0.1002	0.0914	0.0884	0.0804
*G-K Tau*	0.0048	0.0034	0.0094	0.0045	0.0040	…	0.0046	0.0050	0.0042	0.0038	0.0032
*U*	0.0038	0.0027	0.0074	0.0038	0.0033	…	0.0035	0.0040	0.0034	0.0031	0.0026
$\lambda$	0.0340	0.0281	0.0603	0.0080	0.0181	…	0.0044	0.0316	0.0147	0.0316	0.0258

The weights for the $I-MFCESF$ are calculated by using intuitionistic membership grades as in Table 8.

**Table 8:** Weights of the $\mathrm{I}-\mathrm{MFCESF}$ for 3 $\times$ 4 contingency table

Categorical effect size methods	3 $\times$ 4	
	${I-MFCESF}_{1}$	${I-MFCESF}_{2}$
$Cramer{^\prime}s v$	0.1541	0.1699
$Pearson{^\prime}s c$	0.2722	0.0161
$Tschuprows{^\prime}T$	0.2496	0.0677
$Cohen{^\prime}s w$	0.2708	0.0181
$G-K Tau$	0.0155	0.2439
$U$	0.0182	0.2425
$\lambda$	0.0197	0.2417
*MAPE*	0.6656	**0.2753**

Significant values are in [bold].

Table 8 demonstrates that two functions are computed by multiplying each method with their respective weights. In the case of *I-MFCESF*, the weights are determined using intuitionistic membership grades. The functions of the proposed method are derived using the equations provided in Eqs. (21, 22).

21 $$\begin{aligned} I - MFCESF_{1 } & = Cramer^{\prime}s v \times 0.1541 + Pearson^{\prime}s c \times 0.2722 + Tschuprows^{\prime}T \times 0.2496 \\ & \;\;\; + Cohen^{\prime}s w \times 0.2708 + G - K Tau \times 0.0155 + U \times 0.0182 \\ & \;\;\; + \lambda \times 0.0197 \\ \end{aligned}$$

22 $$\begin{aligned} I - MFCESF_{2 } & = I - MFCESF_{best } = Cramer^{\prime}s v \times 0.1699 + Pearson^{\prime}s c \times 0.0161 \\ & \;\;\; + Tschuprows^{\prime}T \times 0.0677 + Cohen^{\prime}s w \times 0.0181 + G - K Tau \times 0.2439 \\ & \;\;\; + U \times 0.2425 + \lambda \times 0.2417 \\ \end{aligned}$$

According to Table 8, it is evident that ${I-MFCESF}_{2 }$ exhibits the lowest *MAPE*. Therefore, ${I-MFCESF}_{2}$ is identified as the best *I-MFCESF*. The *MAPE* of the methods are computed and presented in Table 9 to assess the performance of the proposed method.

**Table 9:** MAPE and BİAS values of the proposed and selected effect size methods for $3\times 4$ contingency tables

Categorical effect size methods	3 $\times$ 4	
	*MAPE*	*BİAS*
$Cramer{^\prime}s v$	0.5650	− 0.0433
$Pearson{^\prime}s c$	0.7357	− 0.1212
$Tschuprows{^\prime}T$	0.5917	− 0.0639
$Cohen{^\prime}s w$	0.7391	− 0.1237
$G-K Tau$	2.3570	0.0277
$U$	3.1442	0.0304
$\lambda$	0.5296	− 0.0062
*I-MFCESF*	**0.2753**	− **0.0032**

Significant values are in [bold].

**Figure 2:**
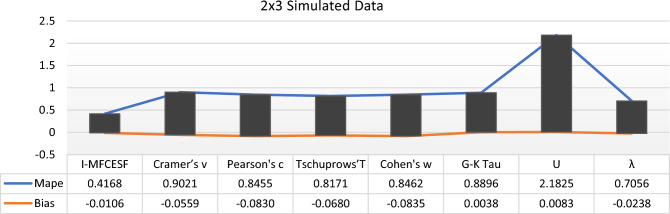
MAPE and Bias values of the *I-MFCESF* and effect size methods for $2\times 3$ simulated data.

**Figure 3:**
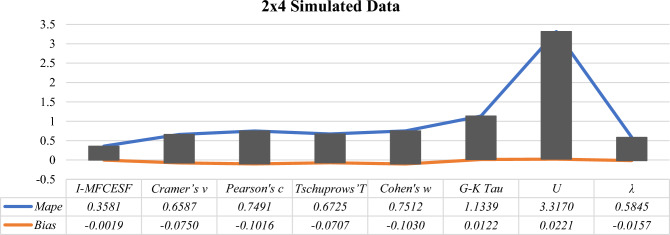
MAPE and Bias values of the *I-MFCESF* and effect size methods for $2\times 4$ simulated data.

**Figure 4:**
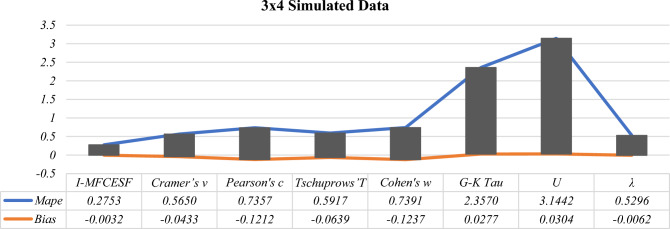
MAPE and Bias values of the *I-MFCESF* and effect size methods for $3\times 4$ simulated data.

Based on the information provided in Table 9, it is evident that the *I-MFCESF* method outperforms the individual categorical ES methods in terms of MAPE. The *I-MFCESF* bias value was determined as respectively. − 3.2 %. Because bias is between ±10%, the accuracy of the proposed method is also sufficient in terms of bias. Figures 2, 3 and 4 illustrate the *MAPE* and Bias values of the proposed methods and selected methods for various contingency tables.

### Real-world categorical dataset for 2 $\times$ 3, 2 $\times$ 4 and 3 $\times$ 4 contingency tables

The first dataset contains 34 variables; 33 of which are categorical and one of them is numerical. There are 366 observations in the dataset. The dataset is a related to the differential diagnosis of erythematous-squamous diseases. The data is taken from the UCI Machine Learning Repository database. It can be open accessed via (https://archive.ics.uci.edu/ml/datasets/Dermatology). The “family history”, “eosinophi”, and “erythema” variables in the “Dermatology” dataset are used. In the dataset, the family history feature has the value “1” if any of these diseases has been observed in the family, and “0” otherwise. Eosinophi has the value “0”” if feature was not present, “1” indicate the relative intermediate values, “2” indicate the largest amount possible. Erythema has the value “0” if feature was not present, “3” indicates the largest amount possible, and “1”, “2” indicate the relative intermediate values. A totally of 1000 different samples with replacements are drawn from the Dermatology dataset. In the proposed method, the input matrix $$(Z)$$ is obtained from the outputs of the calculated categorical ES measures for these samples. Then, the membership grades are obtained by clustering the input matrix with the *IFCM* algorithm. The fuzziness index parameter ($$m)$$ is taken as “2”. Using the membership grades, the weights of each categorical ES method in each cluster are calculated. The next step is to obtain the fuzzy functions by using the weights. There will be as many fuzzy functions as the optimum number of clusters. The optimum cluster number is searched between “2” and “5”, iteratively. Finally, the fuzzy function with the smallest *MAPE* is chosen and the new effect size value is calculated.

#### Family history and Eosinophi variables ($2\times 3$ contingency tables)

“Family history” and “Eosinophi” variables are selected in the Dermatology dataset for $2\times 3$ contingency table. The input matrix $(Z)$ is obtained from outcomes of seven ES measures for these variables. The first five and last five prediction values of the input matrix are summarized in Table 10.

**Table 10:** Input Matrix for family history and eosinophi variables contingency table

[Z]	t_1_	t_2_	t_3_	t_4_	t_5_		t_996_	t_997_	t_998_	t_999_	t_1000_
Cramer’s v	0.2736	0.3333	0.2462	0.5303	0.2270	…	0.2535	0.2611	0.1912	0.2760	0.1920
Pearson’s c	0.2935	0.3393	0.2725	0.4795	0.2579	…	0.2781	0.2839	0.2310	0.2954	0.2316
Tschuprow’s T	0.2582	0.3034	0.2381	0.4595	0.2244	…	0.2435	0.2490	0.1997	0.2600	0.2002
Cohen’s w	0.3071	0.3608	0.2832	0.5464	0.2669	…	0.2896	0.2961	0.2375	0.3092	0.2381
G-K Tau	0.0943	0.1301	0.0802	0.2986	0.0712	…	0.0838	0.0876	0.0564	0.0956	0.0567
U	0.0900	0.0995	0.0889	0.1912	0.0559	…	0.0842	0.0617	0.0489	0.0836	0.0510
$\uplambda$	0.0357	0.0526	0.0555	0.1000	0.0400	…	0.0384	0.0384	0.0357	0.0370	0.0384

The weights for the $I-MFCESF$ are calculated as in Table 11 and ${I-MFCESF}_{1}$ and ${I-MFCESF}_{2}$ are obtained as in Eqs. (23, 24) for Family history and Eosinophi variables.

23 $$\begin{aligned} I - MFCESF_{1 } & = Cramer^{\prime}s v \times 0.2384 + Pearson^{\prime}s c \times 0.2474 + Tschuprows^{\prime}T \times 0.2441 \\ & \;\;\; + Cohen^{\prime}s w \times 0.2451 + G - K Tau \times 0.0144 + U \times 0.0016 \\ & \;\;\; + \lambda \times 0.0090 \\ \end{aligned}$$

24 $$\begin{aligned} I - MFCESF_{2 } & = Cramer^{\prime}s v \times 0.0490 + Pearson^{\prime}s c \times 0.0117 + Tschuprows^{\prime}T \times 0.0186 \\ & \;\;\; + Cohen^{\prime}s w \times 0.0165 + G - K Tau \times 0.2975 + U \times 0.3056 \\ & \;\;\; + \lambda \times 0.3012 \\ \end{aligned}$$

**Table 11:** Weights of the $\mathrm{I}-\mathrm{MFCESF}$ for family history and eosinophi variables

Categorical effect size methods	2 $\times 3$	
	${I-MFCESF}_{1}$	${I-MFCESF}_{2}$
$Cramer{^\prime}s v$	0.2384	0.0490
$Pearson{^\prime}s c$	0.2474	0.0117
$Tschuprows{^\prime}T$	0.2441	0.0186
$Cohen{^\prime}s w$	0.2451	0.0165
$G-K Tau$	0.0144	0.2975
$U$	0.0016	0.3056
$\lambda$	0.0090	0.3012
*MAPE*	0.7162	**0.3196**

Significant values are in [bold].

**Figure 5:**
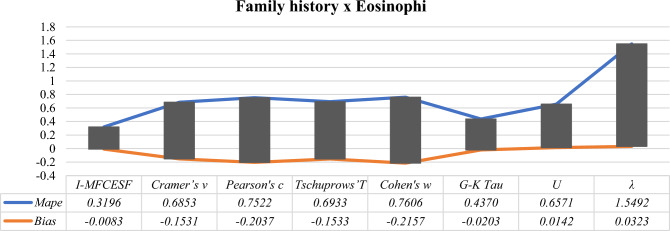
MAPE and Bias values of the *I-MFCESF* and effect size methods for family history and eosinophi variables.

In consideration of Table 11, it is obviously seen that the ${I-MFCESF}_{2 }$ has the lowest *MAPE.* Thus, the best *I-MFCESF* is ${I-MFCESF}_{2}$. Seven methods contribute the performance of the second function. Besides, the sixth method makes the most contribution, but the seventh, fifth, third, fourth, second, and first methods also have an impact on the effectiveness of *I-MFCESF*. The *MAPE* of the methods are computed, and the results are presented in Table 12 to evaluate the performance of the proposed method. Additionally, Fig. 5 provides a visual representation of the *MAPE* and Bias values for the proposed and selected methods specifically for the family history and eosinophi variables.

**Table 12:** MAPE and BİAS values of the proposed and selected effect size methods for family history and eosinophi variables

Categorical effect size methods	MAPE	BİAS
${Cramer{^\prime}s v }$ = 0.0833	0.6853	− 0.1531
${Pearson{^\prime}s c}$ = 0.1106	0.7522	− 0.2037
${Tschuprows{^\prime}T }$ = 0.0936	0.6933	− 0.1533
${Cohen{^\prime}s w}$ = 0.1114	0.7606	– 0.2157
$\mathrm{G}-\mathrm{K Tau}$ = 0.0124	0.4370	− 0.0203
$\mathrm{U}$ = 0.0230	0.6571	0.0142
$\uplambda$ = 0.0012	1.5492	0.0323
I-MFCESF	**0.3196**	**− 0.0083**

Significant values are in [bold].

According to Table 12, it is obviously seen that proposed *I-MFCESF* outperforms other categorical effect size methods in terms of the *MAPE* criterion. Moreover, the bias value of the proposed method is in the range of ± 10%, and it was found to be sufficient in terms of bias. As a result, the new ES value is calculated as 0.020 from Eq. (25).

25 $$\begin{aligned} I - MFCESF_{best } & = 0.0833 \times 0.0490 + 0.1106 \times 0.0117 + 0.0936 \times 0.0186 \\ & \;\;\; + 0.114 \times 0.0165 + 0.0124 \times 0.2975 + 0.0230 \times 0.3056 \\ & \;\;\; + 0.0012 \times 0.3012 = 0.020 \\ \end{aligned}$$

#### Family history and Eryhthema variables ($2\times 4$ contingency tables)

For $2\times 4$ contingency table, “Family history” and “Erythema” variables are selected in the Dermatology dataset. The input matrix of $I-MFCESF$ are obtained from outcomes of seven effect size measures for these variables. The input matrix is summarized in Table 13.

**Table 13:** Input Matrix for family history and eosinophi variables contingency table

[Z]	t_1_	t_2_	t_3_	t_4_	t_5_		t_996_	t_997_	t_998_	t_999_	t_1000_
*Cramer’s v*	0.2067	0.2615	0.1758	0.2475	0.1520	…	0.2708	0.1898	0.3270	0.2917	0.2536
*Pearson’s c*	0.2602	0.2987	0.1188	0.2396	0.2887	…	0.3055	0.2488	0.3462	0.3206	0.2930
*Tschuprow’s T*	0.2047	0.2378	0.1006	0.1875	0.2291	…	0.2437	0.1952	0.2804	0.2571	0.2329
*Cohen’s w*	0.2694	0.3130	0.1197	0.2468	0.3016	…	0.3208	0.2569	0.3690	0.3384	0.3065
*G-K Tau*	0.0726	0.0980	0.0143	0.0609	0.0909	…	0.1029	0.0660	0.1361	0.1145	0.0939
*U*	0.0403	0.0683	0.0104	0.0480	0.0560	…	0.0649	0.0328	0.0439	0.0734	0.0526
$\lambda$	0.0204	0.0169	0.0159	0.0526	0.0357	…	0.0625	0.0164	0.0192	0.0208	0.0244

When the number of clusters was iteratively tried between 2 and 5 to obtain the smallest *MAPE*, it was determined as 3 for this data set. The weights for the $I-MFCESF$ are calculated as in Table 14 and ${I-MFCESF}_{1}$, ${I-MFCESF}_{2}$ and ${I-MFCESF}_{3}$ are obtained as in Eqs. (26–28).

**Table 14:** Weights of the $\mathrm{I}-\mathrm{MFCESF}$ for family history and eryhthema variables

Categorical effect size methods	2 $\times 4$		
	${I-MFCESF}_{1}$	${I-MFCESF}_{2}$	${I-MFCESF}_{3}$
$Cramer{^\prime}s v$	0.0115	0.4854	0.0458
$Pearson{^\prime}s c$	0.0003	0.0036	0.4004
$Tschuprows{^\prime}T$	0.0159	0.4241	0.1290
$Cohen{^\prime}s w$	0.0018	0.0186	0.3939
$G-K Tau$	0.3194	0.0437	0.0186
$U$	0.3301	0.0035	0.0017
$\lambda$	0.3209	0.0211	0.0106
MAPE	0.9709	**0.4767**	0.5943

Significant values are in [bold].

26 $$\begin{aligned} I - MFCESF_{1 } & = Cramer^{\prime}s v \times 0.0115 + Pearson^{\prime}s c \times 0.0003 + Tschuprows^{\prime}T \times 0.0159 \\ & \;\;\; + Cohen^{\prime}s w \times 0.0018 + G - K Tau \times 0.3194 + U \times 0.3301 \\ & \;\;\; + \lambda \times 0.3209 \\ \end{aligned}$$

27 $$\begin{aligned} I - MFCESF_{2 } & = Cramer^{\prime}s v \times 0.4854 + Pearson^{\prime}s c \times 0.0036 + Tschuprows^{\prime}T \times 0.4241 \\ & \;\;\; + Cohen^{\prime}s w \times 0.0186 + G - K Tau \times 0.0437 + U \times 0.0035 \\ & \;\;\; + \lambda \times 0.0211 \\ \end{aligned}$$

28 $$\begin{aligned} I - MFCESF_{3 } & = Cramer^{\prime}s v \times 0.0458 + Pearson^{\prime}s c \times 0.4004 + Tschuprows^{\prime}T \times 0.1290 \\ & \;\;\; + Cohen^{\prime}s w \times 0.3939 + G - K Tau \times 0.0186 + U \times 0.0017 \\ & \;\;\; + \lambda \times 0.0106 \\ \end{aligned}$$

**Figure 6:**
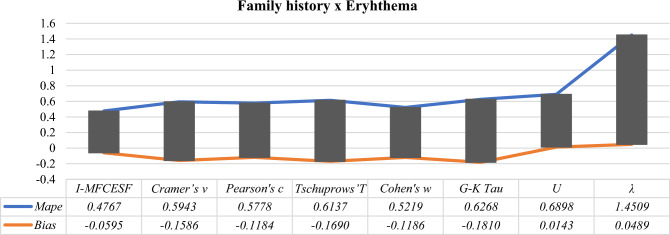
MAPE and Bias values of the *I-MFCESF* and selected methods for family history and eryhthema variables.

According to Table 14, it is seen that the ${I-MFCESF}_{2 }$ has the lowest *MAPE* and the best *I-MFCESF* is ${I-MFCESF}_{2}$. Seven methods contribute to the performance of the proposed method. Besides, the first method makes the most contribution, but the third, fifth, seventh, fourth, second, and sixth methods also have an impact on the effectiveness of *I-MFCESF* respectively. The *MAPE* values of the methods are given in Table 15 to evaluate the performance of the proposed method. Also, Fig. 6 represents the *MAPE* and the Bias values of the proposed and selected methods for family history and eryhthema variables.

**Table 15:** MAPE and BİAS values of the proposed and selected effect size methods for family history and eryhthema variables

Categorical effect size methods	*MAPE*	*BİAS*
$Cramer{^\prime}s v$ = 0.1478	0.5778	− 0.1184
$Pearson{^\prime}s c$ = 0.1706	0.6137	− 0.1690
$Tschuprows{^\prime}T$ = 0.1316	0.5219	− 0.1186
$Cohen{^\prime}s w$ = 0.1732	0.6268	− 0.1810
$G-K Tau$ = 0.0300	0.6898	0.0143
$U$ = 0.0240	1.4509	0.0489
$\lambda$ = 0.0010	2.9487	0.0670
*I-MFCESF*	**0.4767**	**− 0.0595**

Significant values are in [bold].

It is clear from the Table 15 that proposed *I-MFCESF* give very accuracy prediction results for both evaluation criteria MAPE and bias. The MAPE value of the proposed method is better than other categorical effect size methods and the bias value is in the range of ± 10%. Therefore, *I-MFCESF* was found to be sufficient in terms of MAPE and bias. As a result, the new effect size value is calculated as 0.1328 from Eq. (29).

29 $$\begin{aligned} I - MFCESF_{best } & = 0.1478 \times 0.4854 + 0.1706 \times 0.0036 \\ & \;\;\; + 0.1316 \times 0.4241 + 0.1732 \times 0.0186 + 0.0300 \times 0.0437 \\ & \;\;\; + 0.0240 \times 0.0035 + 0.0010 \times 0.0211 = 0.1328 \\ \end{aligned}$$

#### Eosinophi and Eryhthema variables $(3\times 4)$ contingency tables

For $3\times 4$ contingency table, “Eosinophi” and “Eryhthema” variables are selected in the Dermatology dataset. The input matrix of $I-MFCESF$ are obtained from outcomes of seven effect size measures for these variables. The input matrix is summarized in Table 16.

**Table 16:** Input matrix for family history and eosinophi variables contingency table

[Z]	t_1_	t_2_	t_3_	t_4_	t_5_		t_996_	t_997_	t_998_	t_999_	t_1000_
*Cramer’s v*	0.2051	0.0568	0.1385	0.1322	0.1383	…	0.0000	0.0842	0.1069	0.0000	0.0000
*Pearson’s c*	0.3537	0.2505	0.2690	0.2636	0.2989	…	0.1866	0.2633	0.2768	0.2375	0.2023
*Tschuprow’s T*	0.2416	0.1653	0.1975	0.1932	0.2001	…	0.1213	0.1744	0.1841	0.1562	0.1320
*Cohen’s w*	0.3782	0.2587	0.2793	0.2732	0.3132	…	0.1899	0.2729	0.2881	0.2444	0.2066
*G-K Tau*	0.0848	0.0225	0.0096	0.0561	0.0632	…	0.0114	0.0410	0.0333	0.0215	0.0177
*U*	0.0840	0.0458	0.0377	0.0539	0.0521	…	0.0254	0.0582	0.0694	0.0429	0.0377
$\lambda$	0.0588	0.0312	0.0222	0.0357	0.0377	…	0.0196	0.0227	0.0317	0.0182	0.0385

Table 17 is show that the weights are calculated on eosinophi and eryhthema variables. The functions ${I-MFCESF}_{1}$ and ${I-MFCESF}_{2}$, which were created over the weights are given in Eqs. (30) and (31).

30 $$\begin{aligned} I - MFCESF_{1 } & = Cramer^{\prime}s v \times 0.2132 + Pearson^{\prime}s c \times 0.0032 + Tschuprows^{\prime}T \times 0.1232 \\ & \;\;\; + Cohen^{\prime}s w \times 0.0096 + G - K Tau \times 0.2186 + U \times 0.2152 \\ & \;\;\; + \lambda \times 0.2169 \\ \end{aligned}$$

31 $$\begin{aligned} I - MFCESF_{2 } & = Cramer^{\prime}s v \times 0.0446 + Pearson^{\prime}s c \times 0.3405 + Tschuprows^{\prime}T \times 0.2297 \\ & \;\;\; + Cohen^{\prime}s w \times 0.3354 + G - K Tau \times 0.0121 + U \times 0.0211 \\ & \;\;\; + \lambda \times 0.0166 \\ \end{aligned}$$

**Table 17:** Weights of the $\mathrm{I}-\mathrm{MFCESF}$ for eosinophi and eryhthema variables

$$3\times 4$$		
Categorical effect size methods	${I-MFCESF}_{1}$	${I-MFCESF}_{2}$
$Cramer{^\prime}s v$	0.2132	0.0446
$Pearson{^\prime}s c$	0.0032	0.3405
$Tschuprows{^\prime}T$	0.1232	0.2297
$Cohen{^\prime}s w$	0.0096	0.3354
$G-K Tau$	0.2186	0.0121
$U$	0.2152	0.0211
$\lambda$	0.2169	0.0166
MAPE	0.7676	**0.3335**

Significant values are in [bold].

**Figure 7:**
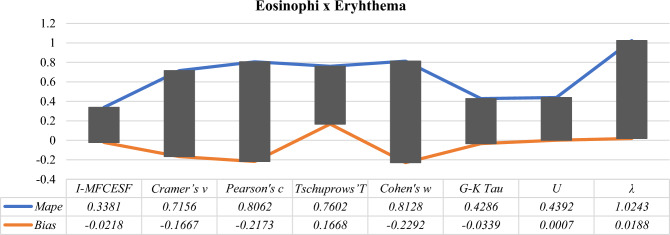
MAPE and Bias values of the *I-MFCESF* and selected methods for eosinophi and eryhthema variables

Considering Table 17, it is clear that regarding the *MAPE* criterion, ${I-MFCESF}_{2 }$ function the best prediction performance for this contingency table. The most contributed performance of the proposed method is $Pearson{^\prime}s c$. Also, other selected methods have smaller impact on the performance of the best function. Figure 7 represents the *MAPE* and the *Bias* values of the selected and proposed methods for eosinophi and eryhthema variables.

Table 18 lists the performances of selected and proposed method. It is obvious by looking at the *MAPE* and the Bias values of the methods that the best performance is produced by the proposed method. The bias value of the proposed methods is in the range of ± 10%, and the *MAPE* value of the proposed method is the lowest according to other effect size methods. Finally, new effect size value is calculated by using Eq. (32).

32 $$\begin{aligned} I - MFCESF_{best } & = 0.0190 \times 0.0446 + 0.1522 \times 0.3405 + 0.0910 \times 0.2297 \\ & \;\;\; + 0.1541 \times 0.3354 + 0.0177 \times 0.0121 + 0.0128 \times 0.0211 \\ & \;\;\; + 0.0162 \times 0.0166 = 0.1260 \\ \end{aligned}$$

**Table 18:** MAPE and BİAS values of the proposed and selected effect size methods for eosinophi and eryhthema variables

Categorical effect size methods	*MAPE*	*BİAS*
$Cramer{^\prime}s v$ = 0.0190	0.9246	− 0.0677
$Pearson{^\prime}s c$ = 0.1522	0.7984	− 0.2571
$Tschuprows{^\prime}T$ = 0.0910	0.6826	− 0.1401
$Cohen{^\prime}s w$ = 0.1541	0.8089	− 0.2760
$G-K Tau$ = 0.0177	0.5041	− 0.0012
$U$ = 0.0128	0.3782	0.0105
$\lambda$ = 0.0162	0.7490	− 0.0177
*I-MFCESF*	**0.3335**	− **0.0370**

Significant values are in [bold].

## Conclusion

The significant two key points of the study can be highlighted as follows. The first, a new approach categorical effect size method based on the *IFCM* and *MFF* is used to ensemble seven different categorical effect size measures. Thus, instead of depending on a single categorical effect size method, seven categorical effect size methods are aggregated for more reliable and accurate outcomes. The second, *I-MFCESF* is an adaptive method that adjust itself based on the given dataset. Some advantages of I-MFCESF are below:

The proposed method incorporates seven different categorical effect size measures that are proposed under various conditions. In the literature, $Cramer{^\prime}s v$*, *$Pearson{^\prime}s c$*, *$Tschuprows{^\prime}T$*, *$Cohen{^\prime}s w$*, *$G-K Tau$*,* $U$ and $\lambda$ effect size measures are most used to $r\times c$ contingency tables. The interpretation ranges of these methods are in the same scale. Thus, these techniques are selected for the proposed method.

$IFCM,$ in which the hesitancy of an object belonging to a cluster with a degree of membership valueis taken into consideration, is used to improve the performance of the proposed method to obtain more accurate results.

$I-MFCESF$ is gathered the information of selected effect size measures in functions by considering their accuracy performances for a dataset. For example, for a given dataset, the X method may perform better than the Y method, while in another dataset, the Y method may perform better than the X method. In this case, the weight of the X method will be higher in the best in the first dataset, while the weight of Y method in the best function will be higher in the second dataset. For this reason, the proposed method has adaptive properties.

$I-MFCESF$ is usually select the best effect size measures with a higher weight in terms of *MAPE* among seven measures.

To demonstrate the performances of the proposed method, we generate two randomly independent categorical variables for *N = 1000* sample and *t = 1000* repeat. Besides, we have investigated Dermatology real-world dataset which are taken from the UCI Machine Learning Repository database. According to the simulation results, *MAPE* was obtained as 0.4168 with a bias of − 0.0106 for the 2 × 3 contingency table, 0.3581 with a bias of − 0.0019 for the 2 × 4 contingency table, and 0.2753 with a bias of − 0.0032 for the 3 × 4 contingency table. The results obtained from the real data, on the other hand, were 0.3196 *MAPE* with a bias of − 0.0083 for the 2 × 3 contingency table, 0.4767 *MAPE* with a bias of − 0.0595 for the 2 × 4 contingency table, and 0.3335 *MAPE* with a bias of − 0.0370 for the 3 × 4 contingency table. Both the simulation study and the applications on the real data set showed us that; the proposed method can predict the results better than the other effect size measures in terms of *MAPE* and bias values. The *MAPE* value of the proposed method was found to be lower in all the application results compared to the other methods, and the bias value was in the range of ± 10%. From the results we can claim that *I-MFCESFs* improve prediction accuracy by combining different effect sizes results. The limitation of the study can be identified as the fact that the performance of the proposed method is affected by the performance of a clustering algorithm. Although, *IFCM* accounts for the hesitancy of an object to be belong to a cluster, it does not consider the outliers in the dataset. In this sense, possibilistic fuzzy clustering algorithm, that accounts for the outliers, can be adapted in *MFF.* This scenario is left for the future study. Therefore, as a future research direction, we plan to combine the effect size measures used for different types of variables and utilize possibilistic fuzzy c-means. Also, to improve the performance of the proposed method, different categorical effect size measures can be included in *MFF*.

## Data Availability

The real dataset are taken from UCI Machine Learning Repository database. It can be open accessed via (https://archive.ics.uci.edu/ml/datasets/Dermatology). The simulated dataset during the current study is available from the corresponding author on reasonable request.
